# Supplementary material for: Detection and Characterization of Estrogen Receptor α Expression of Circulating Tumor Cells as a Prognostic Marker
Source: Cancers (Basel). 2022 May 25;14(11):2621. doi: 10.3390/cancers14112621 (PMC9179654; doi:10.3390/cancers14112621)
Supplement: Supplementary file 1 [file cancers-14-02621-s001.zip › cancers-1681805 supplementary.pdf]

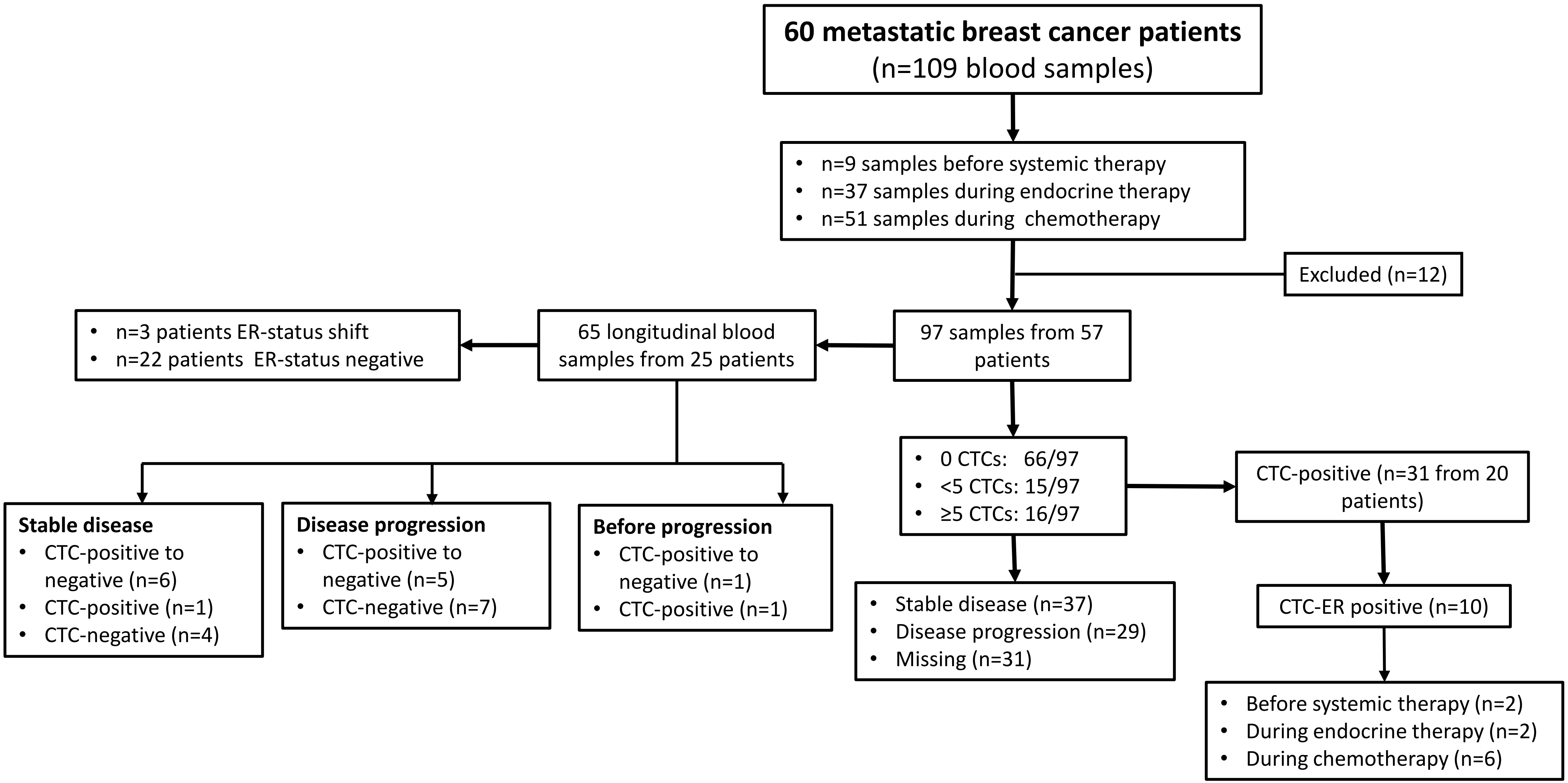

Figure S1. Flow diagram of patient recruitment, exclusions, and CTC status in metastatic breast cancer patients.

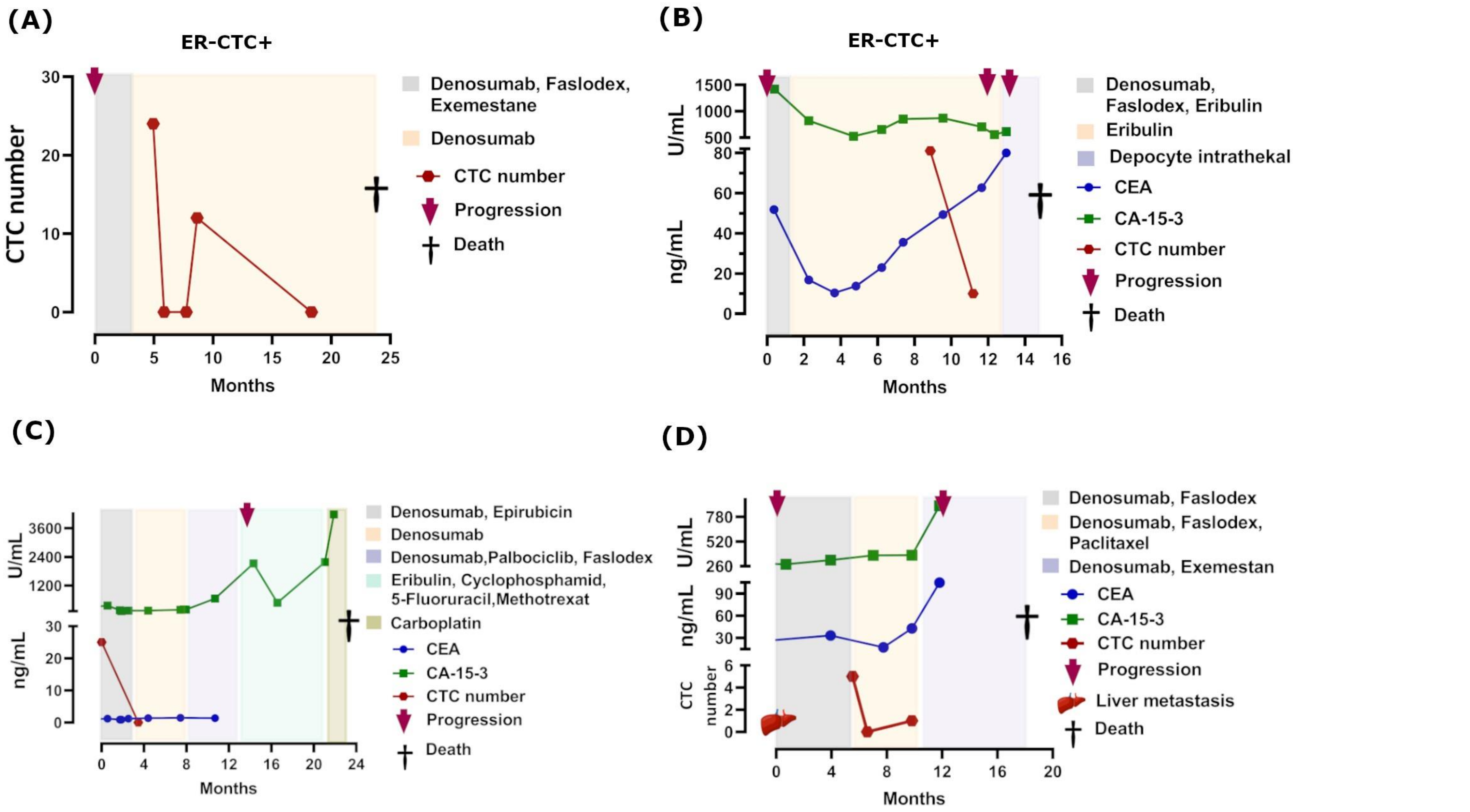

Figure S2. Longitudinal enumeration of CTC in metastatic breast cancer patients. Monitoring of CTC-status in four patients (A–D) before and during progression of disease until death. In addition to the systemic therapy, the concentration of the tumor markers CEA and CA15.
